# Bovine Collagen Peptide Improves Hypoxia Tolerance and Anti‐Fatigue Capacity in Hypobaric Hypoxic Environments: A Combined Animal and Human Study

**DOI:** 10.1002/fsn3.70278

**Published:** 2025-05-15

**Authors:** Rui Zhang, Zi‐Xian Fu, Jun‐Bin Xiong, Wei‐Hong Guo, Wen‐Pu Shi, Bin Jia, Jun‐Ling Shi, Da‐Chuan Yin

**Affiliations:** ^1^ Key Laboratory for Space Bioscience and Biotechnology, School of Life Sciences Northwestern Polytechnical University Xi'an Shaanxi People's Republic of China

**Keywords:** anti‐fatigue, antioxidant, bovine collagen peptide, high‐altitude simulation, hypobaric hypoxia, submaximal exercise test

## Abstract

Peptides are recognized in functional foods for their biological activities. This study investigated the amino acid composition, physicochemical properties, in vitro antioxidant activity, and physiological effects in vivo of bovine collagen peptide (BCP) under hypoxic conditions. Amino acid analysis showed glycine, proline, and glutamic acid as the predominant components. Dynamic light scattering characterized its particle size distribution, while fourier transform infrared spectroscopy (FT‐IR) confirmed its peptide structure. In vitro assays demonstrated significant antioxidant activity of BCP. A two‐stage approach was used to examine BCP's effects on hypoxia tolerance and fatigue reduction under hypobaric hypoxic (HH) conditions. In the first stage, mice were given BCP for 5 days and tested using the rotarod assay at a simulated altitude of 4000 m. The BCP group exhibited an 8‐fold increase in rotarod time compared to the control group (*p* < 0.05), indicating improved hypoxia tolerance and anti‐fatigue capacity. In a human trial at a simulated altitude of 3600 m, 5 days of BCP supplementation led to a 4.7% increase in resting SpO_2_ (*p* < 0.05) and a 15.45% decrease in heart rate (HR) (*p* < 0.05). Submaximal exercise test revealed a 61.54% improvement in hypoxia tolerance (*p* < 0.05) and a twofold increase in anti‐fatigue capacity (*p* < 0.001). This study provides both animal and human evidence supporting the benefits of BCP in HH conditions, suggesting its potential as a functional ingredient for athletes, fitness enthusiasts, and high‐altitude travelers.

## Graphical abstract

This study represents the first demonstration that BCP can significantly enhance hypoxia tolerance and anti‐fatigue capacity under hypobaric hypoxia conditions, as confirmed by both animal and human experiments.

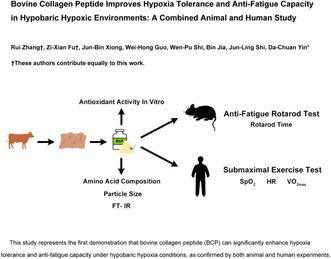

**Abbreviations**
ASL: Above Sea Level
BCP: Bovine Collagen Peptide
BW: Body Weight
CK: Control
ECG: Electrocardiograph
FT‐IR: Fourier Transform Infrared Spectroscopy
HCT: Hematocrit
HD: High dose
HGB: Hemoglobin
HH: Hypobaric Hypoxic
HR: Heart Rate
LD: Low dose
MD: Medium dose
RBC: Red blood cell count
ROS: Reactive oxygen species
SD: Standard Deviation
SpO_2_
: Blood Oxygen Saturation
VO_2max_
: Maximum Oxygen Uptake

## 1 Introduction

Due to reduced atmospheric pressure and lowered oxygen levels, the high‐altitude environment presents unique physiological challenges that cause a range of health problems. People traveling to high altitudes may experience acute altitude sickness, manifested by headache, nausea, and fatigue due to decreased oxygen levels (Luks and Hackett 2022). In severe cases, the condition may progress to cerebral edema or pulmonary edema (Basnyat and Murdoch 2003). In addition, long‐term exposure to high‐altitude areas can lead to a decrease in immune function (Wu et al. 2023), an increase in cardiac workload (Tsao et al. 2021), and a temporary decline in athletic capacity (Flaherty et al. 2016), all of which pose significant health threats. To help counteract the effects of the hypobaric hypoxic (HH) environment, functional foods can be an attractive solution as it is a very mild approach.

As a natural nutritional supplement, functional foods derived from different kinds of bioactive ingredients can relieve fatigue or enhance the body's tolerance to a hypoxic environment through a variety of physiological pathways. The research of plant‐derived anti‐fatigue compounds has been extensive, ranging from common crops such as corn and wheat to medicinal plants like ginseng and yellow extract, each of which demonstrates promising anti‐fatigue effects. Feng et al. (2022) showed that corn polypeptides can significantly prolong the swimming time of mice, effectively reduce the serum urea nitrogen level, increase the storage of muscle and liver glycogen, and alleviate the damage caused by exercise to muscle cells. Zhang et al. (2025) pointed out that ginseng stem and leaf saponins can significantly prolong the swimming time to exhaustion in mice, effectively alleviate fatigue by reducing energy consumption, alleviating oxidative stress damage, and inhibiting the accumulation of harmful metabolites. Compared with plant‐derived peptides, animal peptides demonstrate higher bioavailability owing to their structural similarity to human proteins, enabling them to be more efficiently degraded and absorbed by intestinal enzymes. For example, short peptides extracted from red deer ( *Cervus elaphus* ) blood can significantly extend the swimming time of mice (Lv et al. 2021). Ma et al. (2024) found that sheep skin collagen peptides promote energy metabolism through the AMPK/PGC‐1α axis and reduce oxidative damage through the NRF2/HO‐1 axis, thereby enhancing anti‐fatigue ability. Aquatic organisms evolved in the extreme hypoxic environment and may have formed proteins and peptides that enhance oxygen‐binding ability. Such as Mao et al. found that Antarctic krill ( *Euphausia superba* ) oligopeptide can improve swimming endurance in mice, maintain blood sugar and glycogen levels, reduce lactate dehydrogenase and creatine kinase leakage, enhance muscle ATPase activity, and regulate energy metabolism by activating the AMPK pathway (Mao et al. 2024). Zhao et al. pointed out that croceine croaker ( *Pseudosciaena crocea* ) swim bladder peptide can prolong swimming time, increase glycogen reserve, reduce blood urea nitrogen, lactic acid, and MDA levels, and enhance lactate dehydrogenase activity to remove excess lactic acid and delay the development of fatigue (Zhao et al. 2016). In addition, microbially derived peptides have gradually attracted public attention. Jiang et al. (2018) found that *Tuber melanosporum* peptides can increase the levels of ATP and glycogen in the liver and muscles of mice, reduce serum lactic acid and lactate dehydrogenase, and play an anti‐fatigue role by regulating oxidative stress, energy metabolism, and hormone levels. These foods are effective in relieving fatigue by providing energy, reducing oxidative stress, and promoting muscle repair.

As an important class of ingredients in functional foods, peptides have antioxidant effects (Pownall et al. 2010; Wang et al. 2022, 2025), anti‐fatigue effects (Nakagawasai et al. 2021), regulate the immune system (Fajardo‐Espinoza et al. 2021), and enhance the body's immunity. Bovine collagen peptide (BCP) is a bioactive molecule extracted from bovine collagen, which is believed to have significant health benefits among peptides. However, it remains unclear whether it helps improve hypoxia tolerance and reduce fatigue in the HH environment.

This study systematically investigated the amino acid composition, particle size distribution, FT‐IR spectral characteristics, and antioxidant activity in vitro of BCP. Furthermore, we validated the effects of BCP on enhancing hypoxia tolerance and anti‐fatigue ability under HH conditions through animal experiments and human trials. In the field of anti‐fatigue research, although human trials can give more reliable results, animal experiments are still common practice; therefore, many studies on anti‐fatigue effects lack human trial data. In order to obtain more comprehensive and confident results, we conducted both animal and human trials. The purpose of this study is to validate the application of BCP as a functional food ingredient in high‐altitude environments. The results of this study will provide useful information for the development of targeted nutritional supplements to optimize training performance and athletic capacity for athletes, fitness enthusiasts, mountaineering enthusiasts, journalists, and medical professionals.

## 2 Materials and Methods

### 2.1 Materials and Instruments

The BCP derived from bovine hide and the placebo (milk powder) were purchased from Shaanxi BaiChuanKangZe Biological Technology Co. Ltd. in China. The BCP exhibited a protein content of 98%, while the milk powder contained a protein content of 38.6% (simulating daily nutritional supplementation).

Heart rate (HR) and blood oxygen saturation (SpO_2_) were measured using PC‐80D ECG monitors (LiKang Biomedical Technology Holdings Co. Ltd., China) under HH environmental conditions.

### 2.2 Experiment Reagents

The total antioxidant capacity assay kit was procured from Beyotime Biotechnology Company, China. 2,2‐Diphenyl‐1‐picrylhydrazyl (DPPH) radical and hydroxyl radical scavenging capacity were assessed using commercial assay kits (Nanjing Jiancheng Bioengineering Institute, China). Other reagents are analytical‐grade reagents.

### 2.3 Amino Acid Composition Analysis

The amino acid composition of BCP was determined using a Biochrom 30+ amino acid analyzer (Biochrom Ltd., UK). After the sample preparation, the individual amino acids were separated and detected using ion exchange chromatography. The experimental results, expressed as mass percentages, were used to assess the relative abundance and compositional characteristics of different amino acids in BCP.

### 2.4 Particle Size

The particle size of BCP was determined by the dynamic light scattering technique. The hydrated BCP dispersion was characterized for average hydrodynamic diameter and particle size distribution using a Zetasizer Nano ZS analyzer (Malvern Panalytical, UK). The results were reported in terms of the average particle size and polydispersion index (PDI).

### 2.5 Ft‐IR

The structural characteristics of BCP were analyzed using a FT‐IR spectrometer (Nicolet iS20, Thermo Fisher Scientific, USA). The sample was mixed with anhydrous potassium bromide powder in an appropriate ratio and pressed into a transparent pellet. The infrared absorption spectrum was recorded over the wavenumber range of 4000–400 cm^−1^. These experimental data were utilized to analyze the primary chemical bonds, functional groups, and secondary structural features of the BCP.

### 2.6 Antioxidant Activity In Vitro

#### 2.6.1 Total Antioxidant Capacity

Trolox is a substance similar to vitamin E that has a similar antioxidant capacity to vitamin E and can be used as a reference for the total antioxidant capacity of other antioxidants. When Trolox is used as a standard for total antioxidant capacity testing, the antioxidant capacity of a sample can be expressed by Trolox‐equivalent antioxidant capacity. The total antioxidant capacity of BCP was evaluated using a total antioxidant capacity assay kit (Beyotime Biotechnology, China).

#### 2.6.2 DPPH Radical Scavenging Capacity

DPPH is a stable free radical that exhibits a strong purple color, which is reduced when it reacts with antioxidants. As an antioxidant, BCP neutralizes the DPPH radical by donating electrons or hydrogen atoms, leading to a reduction in the intensity of the purple color. The scavenging activity was quantified by measuring the absorbance at 517 nm, with a decrease in absorbance corresponding to an increase in radical scavenging capacity. The DPPH radical scavenging capacity of BCP was evaluated using a commercial assay kit (Nanjing Jiancheng Bioengineering Institute, China).

#### 2.6.3 Hydroxyl Radical Scavenging Capacity

The Hydroxyl radicals (·OH) are highly reactive species that can cause oxidative damage to cellular components. The Fenton reaction is the most common chemical reaction that produces hydroxyl radicals, and the amount of H_2_O_2_ is proportional to the amount of ·OH produced by the Fenton reaction. After an electron acceptor is given, Griess reagent is used to develop color and form a red substance, whose color is proportional to the amount of·OH. For detailed operational procedures, refer to the official manual provided by the Nanjing Jiancheng Bioengineering Research Institute in China.

### 2.7 Animals

The SPF grade 5 week‐old male Kunming species mice were purchased from the Laboratory Animal Center, Department of Medicine, Xi'an Jiaotong University (License No. SCXK (Shaanxi) 2023‐002). The mice were housed under controlled conditions with a constant ambient temperature of 25°C, a light–dark cycle of 12 h each, and maintained at a humidity level between 35% and 50%. Adequate food and water were provided ad libitum to the mice. All animal experiments strictly adhered to international ethical guidelines and received approval from the Medical and Experimental Animal Ethics Committee of Northwestern Polytechnical University.

The dose selected in this study was determined based on the results of early research and preliminary dose range screening experiments. Specifically, the mice were given low dose (LD, 0.2 g/kg BW), medium dose (MD, 0.7 g/kg BW), and high dose (HD, 1 g/kg BW) BCP for five consecutive days, followed by an exhaustive loaded swimming test and a normobaric hypoxia test. The results showed that the medium dose (0.7 g/kg BW) showed the best effects in prolonging the survival time of hypoxia and improving physical endurance, with significant differences compared with the control group (Figure S1). Since no adverse effects were observed, this dosage was selected for subsequent formal experiments. In the formal experiment, fourteen Kunming mice were divided into a control group (CK group) and an experimental group (BCP group) (gavage concentration of 0.7 g/kg·BW), with 7 mice in each group, and the weight distribution was comparable. The mice in the BCP group received daily gavage ingestion at a dose equivalent to 1% of their body weight, while the control group received an equal volume of sterile saline. The mice underwent intragastric administration once a day for 5 consecutive days. On the 5th day, following a 30‐min gavage period, the HH chamber was adjusted to simulate an altitude of 4000 m, after which an anti‐fatigue rotarod test was performed in this hypoxic environment.

### 2.8 Anti‐Fatigue Rotarod Test in Mice

For 1 week prior to the start of the test, mice are subjected to instrumental training for 30 min daily. During the training phase, a rotating speed of 30 rpm is set, and each mouse is placed on a rotating bar. As soon as a mouse falls, it is immediately relocated to the rod to ensure familiarity with the operation of the rotating rod. In the anti‐fatigue rotarod test, we adjusted the rotating speed to 40 rpm and recorded the duration for which each mouse remained balanced on the rotarod. Repeat this test three times for each mouse to calculate the average rotarod time. After all tests were completed, the mice were immobilized by injection anesthesia, and then blood samples were taken from their ocular region for comprehensive hematological analysis. After euthanizing the mice following ethical guidelines, major organs, including the liver, heart, spleen, lungs, and kidneys, were carefully harvested, rinsed with ice‐cold saline, and weighed using an electronic analytical balance with a precision of 0.01 mg. The organ weight was recorded, and the organ index was calculated by normalizing the organ weight to the body weight using Equation (1):

(1) Organ Index=Organ Weight/Body Weight×100%

### 2.9 Experimental Facility

In order to avoid the complexity and high cost of participants traveling to high‐altitude areas, we have specially designed a facility to simulate a high‐altitude environment to meet the experimental needs of the HH environment. The facility comprises a plexiglass chamber, a metallic chain, a hand pull‐ring, two pulleys, a rubber base, a barometer, a vacuum pump, and a ventilation valve. By integrating the barometer and vent valve within the plexiglass chamber, real‐time display of air pressure inside the chamber can be achieved while the simulated altitude in the chamber can be estimated by Equation (2). Simultaneously, adjusting the valve enables continuous connection between internal and external air to maintain pressure equilibrium. The facility has an outer diameter of 150 cm, a column height of 170 cm, and a roof radius of 75 cm. In addition, it has a wall thickness of 2 cm.

(2) $$P=P_{0}\times {\left(1-H/44300\right)}^{5.256}$$

*H*, altitude (m); *P*_0_, Standard atmospheric pressure (101.325 kPa).

### 2.10 Participants

We recruited 12 healthy college students aged 18 to 25 years who had no professional training, no history of smoking or alcohol consumption, and who did not exhibit any cardiovascular, hepatic, or renal dysfunction or other underlying health problems. After a detailed understanding of the experimental process and protocol, all participants signed informed consent before the experiment. The participants were randomly assigned to two groups: a placebo group and an experimental group (*n* = 6). The experimental group ingested 4 g of BCP daily, while the control group received an equivalent dose of placebo (milk powder) over 5 days. During the trial period, to ensure the precision and dependability of the findings, all participants strictly adhered to a standardized diet specifically designed to mitigate any potential influence of dietary variations on the results while refraining from consuming sports beverages or other nutritional supplements.

### 2.11 Human Trial Design

**FIGURE 1:**
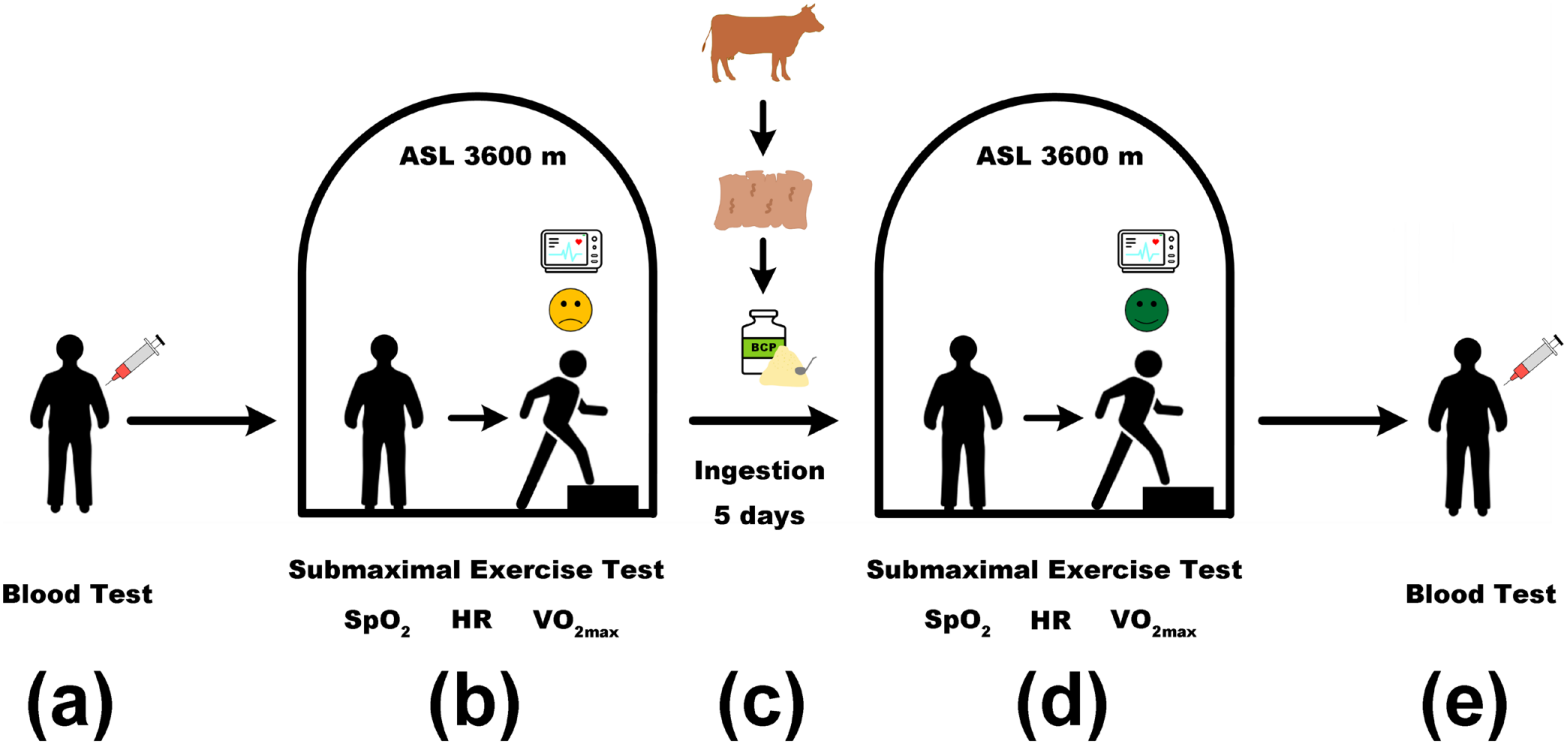
Experimental procedures for the human trial. (a) Blood test before entering the hypobaric hypoxic chamber; (b) Submaximal exercise test at simulated altitude 3600 m in the hypobaric hypoxic chamber, data collected as the control group; (c) 5‐day ingestion, with and without bovine collagen peptide supplementation each day; (d) Submaximal exercise test again at simulated altitude 3600 m in the hypobaric hypoxic chamber, data collected as the experimental group; (e) Blood test after finishing the experiments.

In order to ensure the safety of participants, we chose the altitude of the human trial at 3600 m, rather than at 4000 m (the same altitude as the animal test). In the HH chamber at a simulated altitude of 3600 m, the participants underwent a submaximal exercise test before intake of food, and after intake of food for 5 days. The submaximal exercise test measures the participant's HR and SpO_2_ after repeated steps up and down steps over a period of time. The height of each step is 0.4 m, the stepping speed is 22.5 steps per minute. The stepping time was 5 min for male participants and 3 min for female participants. An ECG recorded each participant's SpO_2_ level and HR during the submaximal exercise test in order to calculate their maximum oxygen uptake (VO_2max_) in a hypoxic environment based on immediate post‐exercise HR data. The VO_2max_ was measured following the methodology described by Wei et al. (2019), with minor modifications. The experimental design is shown in Figure 1.

### 2.12 Blood Collection and Biochemical Analyses

To ensure the accuracy of fundamental hematological and biochemical indicators, as well as to assess the health status of the participants, they were instructed to fast for 8 h before the first supplementation and after the last supplementation, followed by a venous blood draw. We conducted a comprehensive analysis of the complete blood count and evaluated indicators related to liver function, kidney function, lipid metabolism, and glucose metabolism.

### 2.13 Statistical Analysis

All data are presented as mean ± standard deviation (Mean ± SD). Statistical analyses were conducted using GraphPad Prism 8.0 software (GraphPad Software Inc., CA, USA). Intergroup differences were assessed utilizing Student's unpaired t‐test. For comparisons involving more than two groups, a one‐way ANOVA followed by Tukey's multiple comparison test was employed to determine statistical significance. The levels of statistical significance are denoted as follows: *p < 0.05, **p < 0.01, ***p < 0.001, ****p < 0.0001.

## 3 Results

### 3.1 Amino Acid Composition of BCP

**FIGURE 2:**
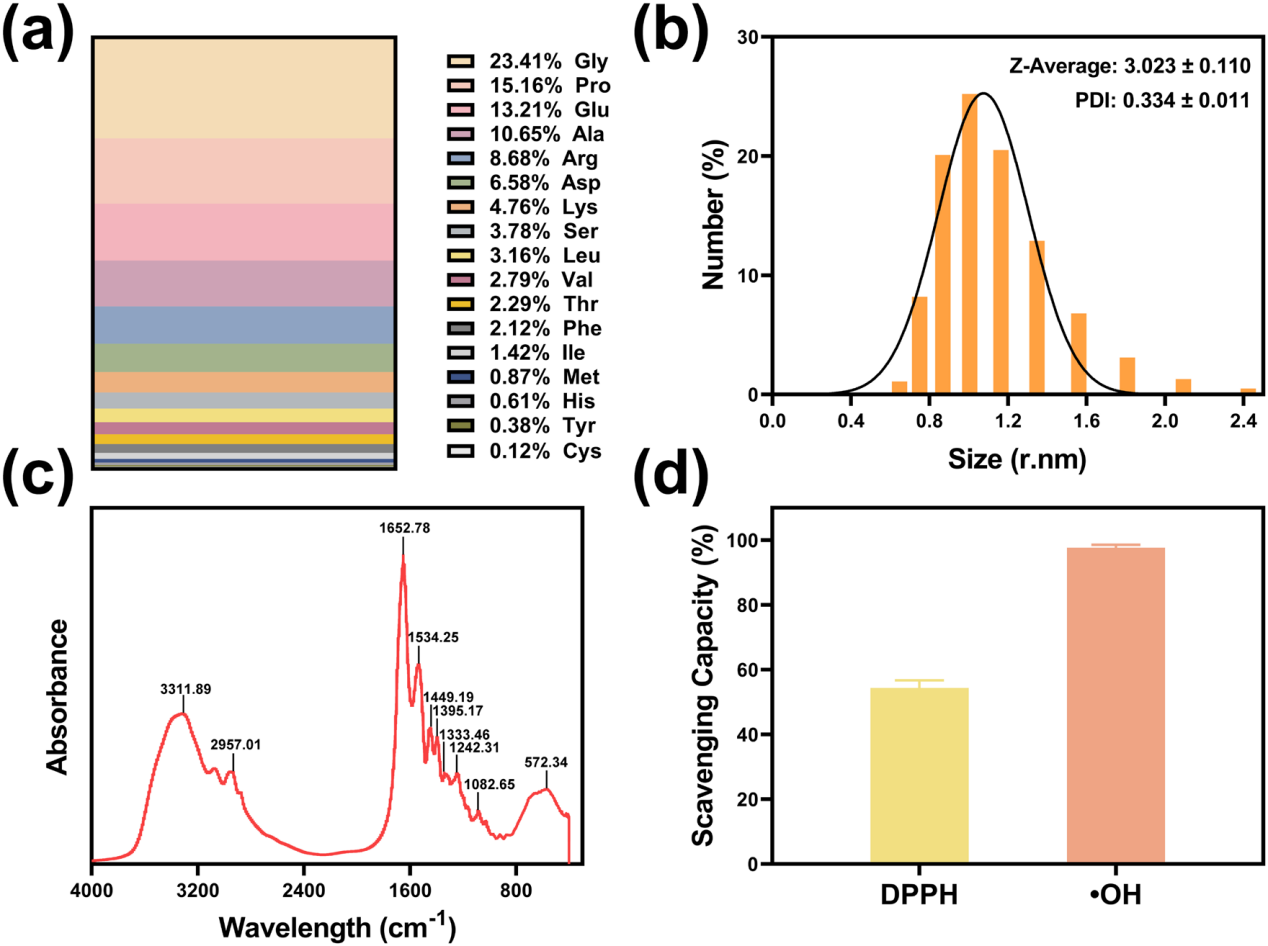
Physicochemical properties of bovine collagen peptide. (a) Amino Acid Composition; (b) Particle Size; (c) FT‐IR; (d) Antioxidant activity in vitro.

Figure 2a and Table S1 offer a comprehensive depiction of the amino acid profile of BCP, detailing both the absolute content (mg/g) and the relative percentage (%). Glycine is the most abundant, with a content of 207.221 mg/g (23.41%), followed by proline at 134.145 mg/g (15.16%) and glutamic acid at 116.948 mg/g (13.21%). Alanine and arginine are also present in significant amounts, with 94.226 mg/g (10.65%) and 76.83 mg/g (8.68%). Aspartic acid contributes 58.255 mg/g (6.58%), while serine is found at 33.502 mg/g (3.78%). Lysine, valine, and leucine are present at 42.13 mg/g (4.76%), 24.724 mg/g (2.79%), and 27.996 mg/g (3.16%). Methionine and cysteine, which are sulfur‐containing amino acids, have relatively low contents, representing 0.87% and 0.12% of the total composition.

### 3.2 Particle Size

The particle size distribution of the BCP was evaluated by Dynamic Light Scattering at 25°C. The result is shown in Figure 2b; the hydrodynamic diameter (Z‐average) of BCP particles was found to be 3.023 ± 0.110 nm, indicating the presence of particles in a nano‐sized range. The PDI, which describes the distribution of particle sizes, was 0.334 ± 0.011, suggesting a relatively narrow distribution of particle sizes. These results suggest that the BCP nanoparticles are of a relatively small and uniform size, which is consistent with the typical characteristics of peptides in aqueous solutions.

### 3.3 Ft‐IR

The FT‐IR spectrum of BCP demonstrated prominent absorption peaks indicative of its chemical composition and structural characteristics (Figure 2c). The strong and broad peak at 3311.89 cm^−1^ corresponds to the N‐H stretching vibration, highlighting the presence of hydrogen bonds and peptide backbone amine groups. The peak at 2957.01 cm^−1^ is attributed to the C‐H stretching vibration, suggesting alkyl or methylene groups in the side chains. The Amide I band at 1652.78 cm^−1^, primarily associated with the stretching vibration of the C=O bond, is a key marker of the peptide backbone and provides insight into secondary structural elements such as α‐helices or β‐sheets. The Amide II band at 1534.25 cm^−1^, caused by N‐H bending and C‐N stretching vibrations, further confirms the presence of peptide bonds. Additionally, the Amide III band at 1242.31 cm^−1^ and other minor peaks, such as those at 1449.19 cm^−1^ and 1082.65 cm^−1^, reflect C‐N, C‐C, and side‐chain‐related vibrations, suggesting the presence of a complex peptide network.

### 3.4 Antioxidant Activity In Vitro

The total antioxidant activity of BCP resulted in an antioxidant capacity of 15.10 ± 1.44 mM Trolox equivalents at a concentration of 10 mg/mL. The results are illustrated in Figure 2d; in the DPPH radical scavenging assay, BCP demonstrated a scavenging rate of 54.37% ± 2.36%, indicating a moderate ability to neutralize DPPH radicals. Additionally, BCP showed a strong hydroxyl radical scavenging capacity of 97.69% ± 0.87% at the same concentration, suggesting a highly effective neutralization of hydroxyl radicals.

### 3.5 Anti‐Fatigue Capacity of Mice in HH Environment

**FIGURE 3:**
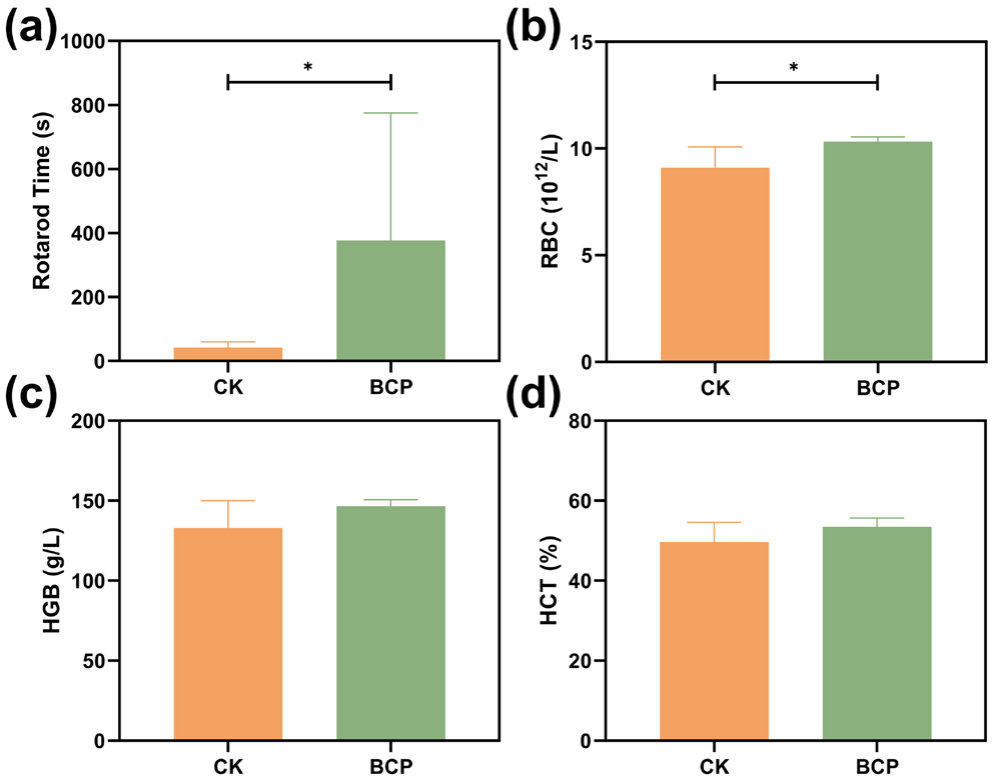
Bovine collagen peptide supplementation helped improve the anti‐fatigue performance of mice in animal experiments. (a) Rotarod time of mice in hypobaric hypoxic chamber; (b‐d) Blood test of mice with and without bovine collagen peptide supplementation. (b) Red blood cell count (RBC); (c) Hemoglobin concentration (HGB); (d) Hematocrit (HCT). Data are expressed as Mean ± SD, *n* = 6. *Compared with the control group, *p* < 0.05.

In an HH environment equivalent to an altitude of 4000 m, mice in the control group exhibited significant discomfort, including closed eyes and decreased activity levels, suggesting symptoms similar to altitude sickness. The anti‐hypoxia and anti‐fatigue capacities of mice were evaluated by the anti‐fatigue rotarod test. The experimental results (Figure 3a) showed that the rotarod time of mice in the BCP group was significantly prolonged, about 8 times that of the control group (*p* < 0.05). Although there were individual differences in the rotarod time of BCP group mice, the overall results still showed that the BCP supplementation significantly improved their tolerance to hypoxia and fatigue.

After 5 days of intragastric feeding, the results of the complete blood cell count in mice are presented in the Table S2. The BCP group exhibited a significant increase in red blood cell count (RBC) (Figure 3b). Although there was no statistically significant difference observed in hematocrit (HCT) and hemoglobin concentration (HGB), mean values were higher than those of the control group and displayed an upward trend (Figure 3c,d). This suggests that erythrocyte production may have been enhanced, leading to improved blood oxygen carrying capacity, energy metabolism, and ultimately alleviating discomfort caused by hypoxic environments while enhancing anti‐fatigue capacity in mice.

The results of organ weight and index are shown in Tables 1 and 2. There was no significant alteration in the heart index of the BCP group, indicating a relatively minor impact on cardiac weight. In contrast, compared to the control group, both liver index and weight were significantly increased in the BCP group, suggesting a protective effect on hepatic function. While spleen index reduced slightly within the BCP group, kidney and lung indices increased; however, these changes did not reach statistical significance.

**TABLE 1:** Effects of bovine collagen peptide on the organ weight of mice (Mean ± SD, *n* = 6).

	Organ weight (g)				
	Heart	Liver	Spleen	Lung	Kidney
CK	0.2042 ± 0.0423	1.6905 ± 0.1546	0.1268 ± 0.0507	0.2277 ± 0.0478	0.2800 ± 0.0535
BCP	0.2088 ± 0.0248	2.2698 ± 0.2653****	0.1196 ± 0.0283	0.2607 ± 0.0625	0.2955 ± 0.0423

**** Compared with the control group, *p* < 0.0001.

**TABLE 2:** Effects of bovine collagen peptide on the organ index of mice (Mean ± SD, *n* = 6).

	Organ index (%)				
	Heart	Liver	Spleen	Lung	Kidney
CK	0.4588 ± 0.0948	3.7944 ± 0.3319	0.2829 ± 0.1083	0.5102 ± 0.1004	0.6276 ± 0.1110
BCP	0.4694 ± 0.0429	5.0943 ± 0.3118****	0.2668 ± 0.0466	0.5830 ± 0.1153	0.6641 ± 0.0803

**** Compared with the control group, *p* < 0.0001.

### 3.6 Analysis of Human Blood Indicators Before and After Experiments in HH Chamber

**FIGURE 4:**
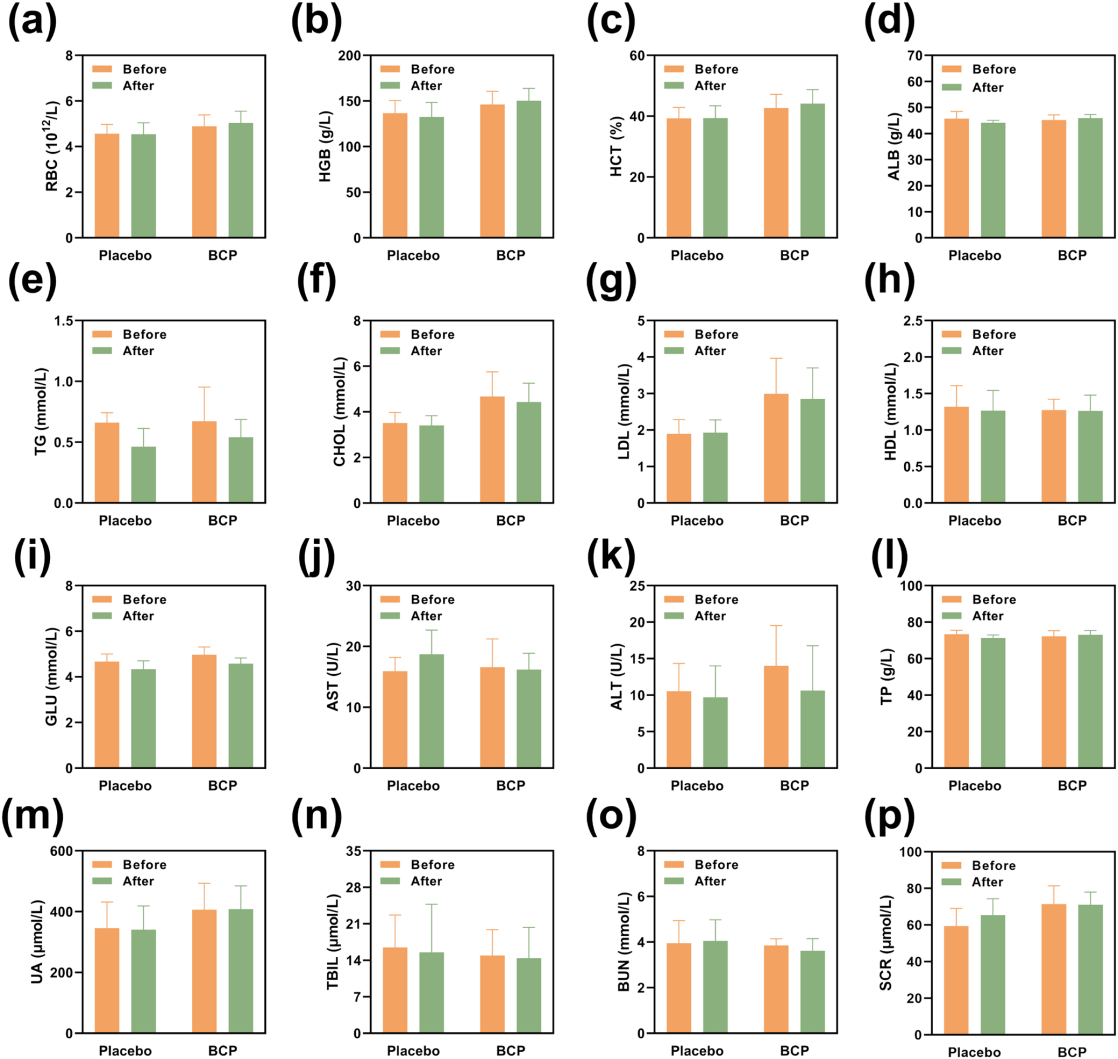
Hematological indicators of the participants before and after human trials. (a) RBC: Red Blood Cell; (b) HGB: Hemoglobin; (c) HCT: Hematokrit; (d) ALB: Albumin; (e) TG: Triglyceride; (f) CHOL: Total Cholesterol; (g) LDL: Low Density Lipoprotein; (h) HDL: High Density Lipoprotein; (i) GLU: Glucose; (j) AST: Aspartate Transaminase; (k) ALT: Alanine Aminotransferase; (l) TP: Total Protein; (m) UA: Uric Acid; (n) TBIL: Total Bilirubin; (o) BUN: Blood Urea Nitrogen; (p) SCR: Serum Creatinine. Data are expressed as Mean ± SD, *n* = 6.

Figure 4 presents a summary of some hematological indicators of participants before and after human experiments in the HH chamber. Refer to the supplementary information for specific human blood indicators (Tables S3–S6). The results showed that all data in the four groups (placebo group, before and after placebo supplementation; BCP group, before and after BCP supplementation) remained within the normal physiological range, suggesting that placebo or BCP supplementation had little effect on blood components and did not adversely affect hematopoiesis, liver function, renal function, glucose metabolism, or lipid metabolism.

After 5 days of BCP supplementation, the RBC increased by 0.15 ± 0.15 × 10^12^/L, HCT increased by 1.42 ± 1.56%, and HGB increased by 4.17 ± 3.87 g/L in the BCP group, while there was little change in the placebo group. It can be seen from the data that the levels of RBC, HCT, and HGB showed an upward trend in the BCP group compared with the placebo group. Serum albumin levels slightly increased in the BCP group (0.78 ± 0.96 g/L) but decreased in the placebo group (−1.57 ± 2.32 g/L). Alanine aminotransferase levels decreased in the BCP group (−5.15 ± 5.34 U/L), surpassing the minimal reduction observed in the placebo group (−0.83 ± 1.90 U/L). Total protein levels increased slightly in the BCP group (0.85 ± 2.12 g/L) but decreased in the placebo group (−2.12 ± 2.32 g/L). In terms of renal function, serum creatinine levels decreased modestly in the BCP group (−0.40 ± 6.20 μmol/L) but increased in the placebo group (6.00 ± 9.93 μmol/L). Triglyceride levels decreased in both groups, with a slightly larger reduction in the BCP group (−0.21 ± 0.25 mmol/L) compared to the placebo group (−0.20 ± 0.21 mmol/L). Fasting blood glucose levels also decreased in both groups, with the BCP group showing a reduction of 0.40 ± 0.24 mmol/L, compared to 0.34 ± 0.30 mmol/L in the placebo group.

### 3.7 Effect of BCP Supplementation on Human Physiological Indicators (SpO_2_ and HR) in HH Environment

**FIGURE 5:**
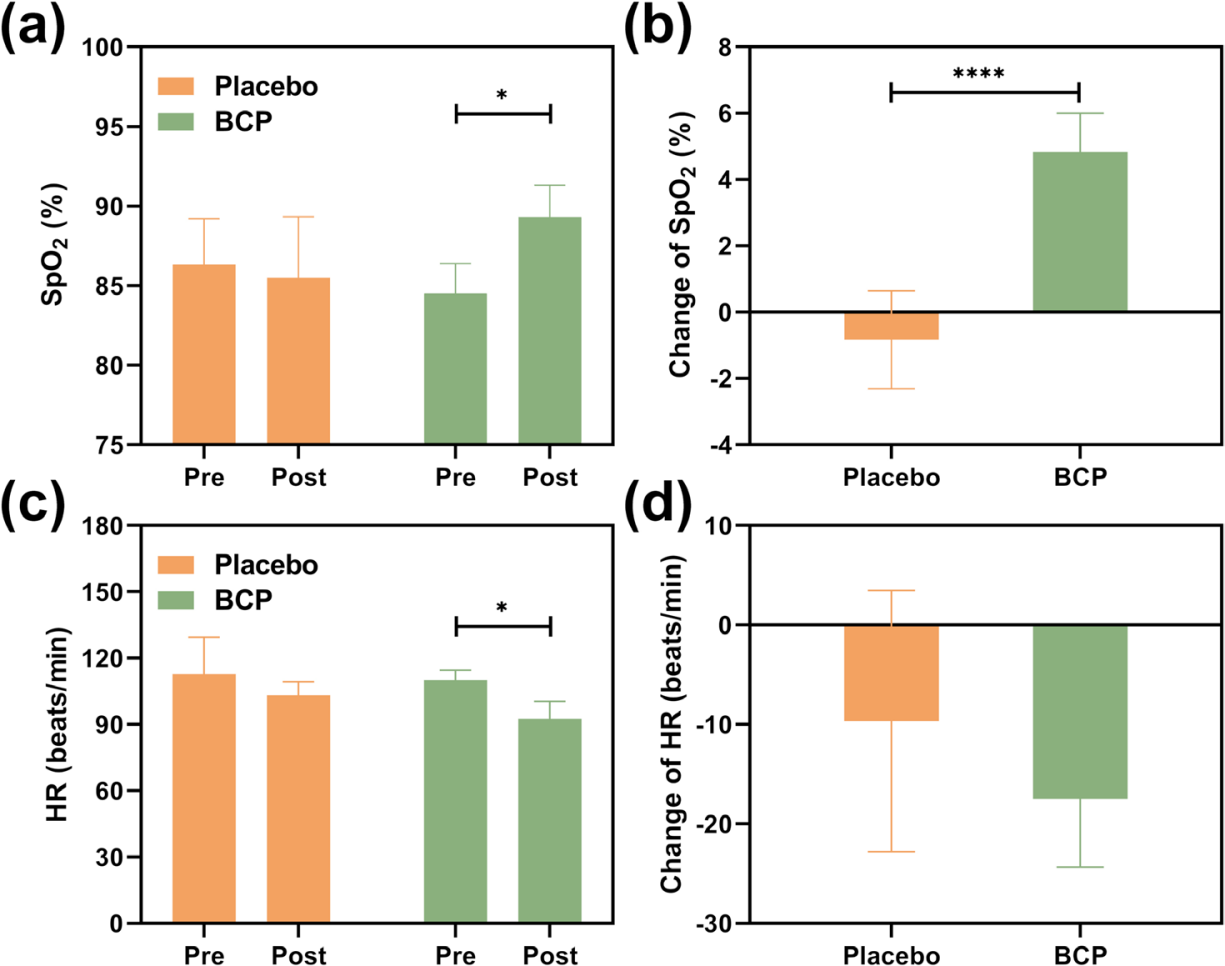
The effect of bovine collagen peptide supplementation on the physiological indicators of the participants at a simulated altitude of 3600 m in a resting state. (a) SpO_2_ values; (b) Change of SpO_2_; (c) Heart rate values; (d) Change of heart rate. Data are expressed as Mean ± SD, *n* = 6, *Compared with the placebo group, *p* < 0.05; ****Compared with the placebo group, *p* < 0.0001.

This study compared the participants' physiological indicators (SpO_2_ and HR) at resting state in the HH chamber before and after BCP supplementation (Figure 5). It was found that (Figure 5a), in an HH chamber simulating an altitude of 3600 m, participants in the placebo group exhibited a baseline SpO_2_ of 86% before ingestion and after ingestion, with no statistically significant difference observed. Participants receiving BCP supplementation demonstrated an increase in SpO_2_ from 85% before ingestion to 89% after ingestion, reflecting a notable enhancement of 4.7% (*p* < 0.05). Furthermore, the SpO_2_ levels in the placebo group remained almost unchanged before and after ingestion. In contrast, the BCP group displayed a substantial increase in SpO_2_ after ingestion, with an average rise of 4% (*p* < 0.0001) as illustrated in Figure 5b.

In the case of HR, the results are presented in Figure 5c. In the HH chamber simulating an altitude of 3600 m, participants in the placebo group exhibited a resting HR of 113 beats/min before supplementation and 103 beats/min after supplementation, with no statistically significant difference observed. Participants in the BCP group demonstrated a resting HR of 110 beats/min before supplementation and a reduction to 93 beats/min after supplementation, reflecting a decrease of 15.45% (*p* < 0.05). The placebo group experienced a decline of 10 beats/min in HR from before to after supplementation, while the BCP group showed a more pronounced decrease of 17 beats/min following supplementation. Both groups exhibited reductions in resting HR at an altitude of 3600 m (Figure 5d), indicating that BCP supplementation significantly mitigated HR elevation under hypoxic conditions.

### 3.8 Effect of BCP Supplementation on Hypoxia‐Tolerance Performance (Anti‐Hypoxic Capacity and Anti‐Fatigue Capacity) Under HH Environment

When the body engages in physical exercise under hypoxic conditions, SpO_2_ levels can significantly decline, resulting in an insufficient oxygen supply to vital organs and tissues. The deficiency may impair the functionality of critical organs such as the heart and brain, while also reducing muscle oxygen availability, thereby affecting muscular efficiency and significantly impairing athletic capacity. Furthermore, it may expedite fatigue onset and increase the risk of sports‐related injuries.

The supplementation of BCP resulted in a notable enhancement in SpO_2_ levels of participants at rest. To thoroughly assess the participants' hypoxia tolerance during hypoxia exercise, we computed the change in SpO_2_ before and after the submaximal exercise test. The percentage increase in tolerance to hypoxia is calculated according to Equation (3):

(3) $$\text{Increase in oxygen tolerance}\;\left(\%\right)=\frac{\left|\Delta {\mathrm{SpO}}_{2\text{after}}-\Delta {\mathrm{SpO}}_{2\text{before}}\right|}{\Delta {\mathrm{SpO}}_{2\text{before}}}\times 100\%$$

The ∆SpO2 after refers to the difference in SpO_2_ levels before and after exercise following supplementation with BCP, while ∆SpO2 before denotes the difference in SpO_2_ levels before and after exercise prior to BCP supplementation.

**FIGURE 6:**
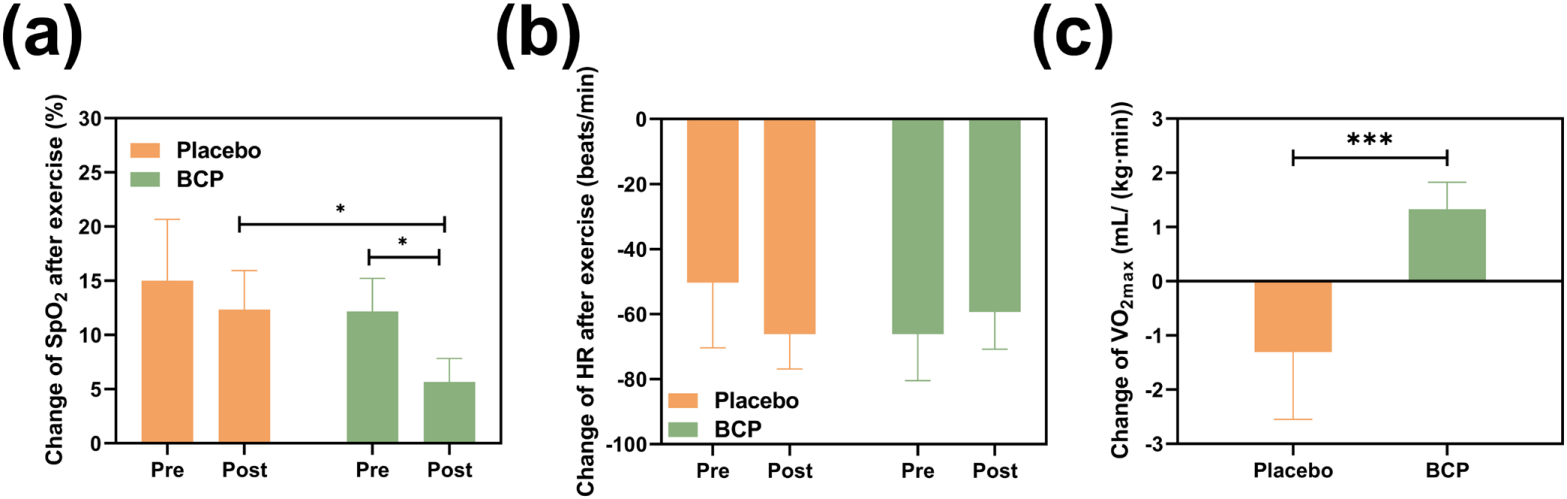
The physiological indicators before and after the submaximal exercise test at the simulated altitude of 3600 m. (a) Changes in SpO_2_ before and after exercise; (b) Changes in heart rate before and after exercise; (c) Changes in VO_2max_. Data are presented as Mean ± SD, *n* = 6. *Indicates a comparison with the control group, *p* < 0.05; ***Indicates a comparison with the control group, *p* < 0.001.

It was calculated that the results, as shown in Table 3 and Figure 6a, before the ingestion, the SpO_2_ of the placebo group participants after stepping was 15% lower than before stepping. After ingestion, the SpO_2_ after stepping was 12% lower than before stepping, and the difference was not significant. Conversely, for participants in the BCP group, after stepping, SpO_2_ was recorded at 72%, reflecting a decrease of 13% compared to their pre‐stepping level of 85%. After ingestion, SpO_2_ after stepping measured at 84%, down from 89%, representing a reduction of only 5%. Notably, supplementation with this additive resulted in significantly higher post‐exercise SpO_2_ levels for the BCP group compared to both their pre‐supplementation values (*p* < 0.01) and those of the placebo group (*p* < 0.05). Furthermore, when comparing changes between groups, there was a marked reduction in SpO_2_ differences before and after exercise within the BCP group relative to placebo. According to these findings, BCP enhances hypoxia tolerance by 61.54%. It is evident that BCP supplementation significantly mitigates changes in SpO_2_ while enhancing physiological adaptability under hypoxic conditions. As shown in Figure 6b, BCP supplementation reduced the difference in HR before and after submaximal exercise test, thereby improving the body's exercise tolerance under a hypoxic environment.

**TABLE 3:** Physiological indicators measured before and after both BCP supplementation and submaximal exercise test.

	Placebo		BCP	
	Pre‐ingestion	Post ingestion	Pre‐ingestion	Post ingestion
Pre‐step SpO_2_ (%)	86 ± 3	86 ± 4	85 ± 2	89 ± 2*
Post‐step SpO_2_ (%)	71 ± 8	73 ± 6	72 ± 3	84 ± 3**^,#^
Pre‐step HR (beats/min)	113 ± 17	103 ± 6	110 ± 4	93 ± 8*
Post‐step HR (beats/min)	163 ± 9	169 ± 8	167 ± 8	161 ± 8
VO_2max_ mL/(kg·min)	40.23 ± 1.81	38.92 ± 1.39	39.03 ± 1.55	40.36 ± 1.45

*Note:* Indicates intragroup comparisons, specifically the difference between the supplemented and non‐supplemented groups; *p < 0.05, **p < 0.01. Indicates intergroup comparisons, referring to the difference between the BCP group and the Placebo group; #*p* < 0.05.

The equations for calculating VO_2max_ and exercise‐induced HR (HR_e_) are as follows (Wei et al. 2019):

(4) $${\mathrm{VO}}_{2\max}=10^{\left(0.438621-0.002626{\mathrm{HR}}_{e}+0.006238\mathrm{BW}\right)*1000/\mathrm{BW}}$$

(5) $${\mathrm{HR}}_{e}=23.2625+0.8758\times {\mathrm{HR}}_{i}$$

Among these indicators, the HR_*i*_ refers to the HR measured at the end of stepping, HR_*e*_ denotes the exercise‐induced HR, and BW represents the bodyweight.

By measuring the HR_*i*_ post‐exercise, we can calculate the HR_*e*_ and subsequently derive the participant's VO_2max_. In evaluating the participant's anti‐fatigue capacity under hypoxic conditions, we can assess changes in VO_2max_ before and after supplementation.

The percentage increase in anti‐fatigue capacity is calculated according to Equation (6):

(6) $$\text{Increase in anti}‐\text{fatigue capacity}\;\left(\%\right)=\frac{\left|{\mathrm{VO}}_{2\max\text{after}}-{\mathrm{VO}}_{2\max\text{before}}\right|}{{\mathrm{VO}}_{2\max\text{before}}}\times 100\%$$

The term “VO_2maxafter_” refers to the post‐supplementation VO_2max_ levels following supplementation of BCP, while “VO_2maxbefore_” represents the pre‐supplementation VO_2max_ levels.

According to the equations, Table 3 illustrates that the VO_2max_ following the placebo group was 1.31 mL/(kg min) lower than before ingestion. In contrast, the VO_2max_ after the BCP group increased by 1.33 mL/(kg min) compared to before ingestion, indicating a twofold enhancement in anti‐fatigue capacity. Figure 6c demonstrates that VO_2max_ before and after ingestion was significantly different between the placebo group and the BCP group (*p* < 0.001). Therefore, supplementation with BCP can substantially improve cardiopulmonary function and enhance the body's efficiency in utilizing oxygen.

## 4 Discussion

This study investigated the amino acid composition, physicochemical properties, antioxidant activity in vitro, and physiological effects in vivo of BCP under hypoxic conditions. A two‐step experimental method combining animal and human trials demonstrated the efficacy of BCP in enhancing anti‐fatigue capacity and hypoxia tolerance under HH conditions. The findings give strong evidence for the physiological benefits of BCP supplementation, providing new insights into functional foods aimed at improving performance and resilience in extreme environments.

The high levels of glycine and proline in BCP support its role in enhancing tissue repair and restoration (Dai et al. 2018), which is crucial for combating oxidative stress caused by hypoxia and high‐intensity exercise. Glycine promotes energy production by participating in the tricarboxylic acid cycle and exhibits anti‐inflammatory properties to alleviate damage caused by hypoxia (Paz‐Lugo et al. 2018). Furthermore, glycine plays a crucial role in the biosynthesis of serum glycocholic acid, sarcosine, heme, and various other biological compounds within the body (Garcia‐Santos et al. 2017). Additionally, it exhibits antioxidant properties that aid in protecting cells from oxidative damage (Huang et al. 2016). Similarly, proline enhances the stability of the extracellular matrix and supports cellular antioxidant mechanisms (Komatsu et al. 2020). Glutamate is an important component that participates in the synthesis of glutathione, a critical antioxidant molecule, thereby reducing oxidative stress under hypoxic conditions. Essential amino acids, including lysine, leucine, and valine, further enhance the bioactivity of BCP. Lysine supports collagen synthesis, promoting effective oxygen utilization in muscle tissue (Tang et al. 2023), while branched‐chain amino acids such as leucine and valine provide direct energy sources and inhibit muscle protein breakdown, reducing exercise‐induced fatigue (Muscella et al. 2024). Additionally, arginine can promote the production of nitric oxide, improving vascular function and oxygen delivery in hypoxic conditions (Lu et al. 2024).

The small particle size suggests that the BCP molecules or aggregates exist in the form of nano‐sized particles. This is consistent with previous studies on collagen peptide, where peptides with molecular weights typically in the range of 500–3000 Da tend to form nanoparticles under certain conditions. The small particle size could potentially enhance the peptide's solubility and absorption (Kwok et al. 2024), facilitating its bioactivity, especially in the context of functional food and therapeutic applications. Additionally, small‐sized particles are known to exhibit better permeability across biological membranes, which could contribute to improved cellular uptake and efficacy in vivo.

The results of this study demonstrate the potent antioxidant properties of BCP. The superior hydroxyl radical scavenging activity suggests that BCP is particularly effective in neutralizing highly reactive free radicals, such as hydroxyl radicals, which are known to cause significant oxidative damage to cellular components (Li et al. 2024). This is noteworthy as hydroxyl radicals play a key role in various pathological conditions, including aging, neurodegenerative diseases, and cardiovascular disorders. The stronger scavenging ability against hydroxyl radicals compared to DPPH radicals highlights the potential of BCP to protect cells and tissues from oxidative stress under more reactive and damaging conditions (Wu et al. 2025). Therefore, BCP's superior performance in the hydroxyl radical scavenging assay suggests it may be particularly beneficial in environments where oxidative stress is intense, such as during physical exertion, exposure to hypoxia, or inflammatory conditions. These findings support the hypothesis that BCP, with its strong antioxidant capacity, could play a significant role in enhancing hypoxia tolerance and anti‐fatigue effects. Reactive oxygen species (ROS) play a pivotal role in the development of oxidative stress (Zheng et al. 2024). Furthermore, oxidative stress is a critical factor contributing to fatigue, particularly under conditions of low oxygen availability. The capacity of BCP to effectively neutralize hydroxyl radicals provides further evidence that it may help mitigate oxidative damage and improve endurance, particularly in hypoxic environments (Zhou et al. 2024). BCP, through its antioxidant properties, could contribute to improved exercise capacity, reduced ROS levels, and reduced fatigue, making it a promising candidate for anti‐fatigue and hypoxia‐tolerance applications.

In animal experiments, mice in the BCP group exposed to a simulated altitude of 4000 m exhibited an 8‐fold increase in rotarod time compared to the control group. This improvement was accompanied by hematological changes, including increased RBC, HCT, and HGB levels. These changes are consistent with the physiological mechanism of hypoxia compensation, in which enhanced erythropoiesis improves oxygen delivery and utilization to support aerobic metabolism (Jin et al. 2024; Park et al. 2018). These results suggest that BCP supplementation can accelerate or amplify the body's natural hypoxia adaptation (Wojan et al. 2023), effectively reducing fatigue and enhancing endurance. The liver index showed the most significant impact of BCP supplementation, exhibiting a statistically significant increased compared to the control group. This rise suggests that BCP has a liver protective effect, possibly related to its antioxidant properties or ability to regulate lipid metabolism. A slight decline in the spleen index may indicate regulated immune function, and the spleen plays a key role in the immune response. The slight increase in lung and kidney indices may reflect an improvement in metabolic efficiency or a reduction in oxidative stress.

Human trials provided additional evidence for these effects. After 5 days of BCP supplementation, participants in a simulated HH environment at 3600 m demonstrated increased resting SpO_2_, decreased resting HR, and improved VO_2max_, indicating better oxygenation and cardiovascular efficiency. During submaximal exercise testing, smaller changes in SpO_2_ and HR in the BCP group indicated reduced physiological stress and improved oxygen utilization. These effects suggest that BCP not only enhances endurance but also helps the body adapt to hypoxic conditions by optimizing oxygen metabolism (Park et al. 2025) and alleviating common altitude‐related symptoms such as palpitations and fatigue.

The observed improvements in oxygen delivery and utilization can be attributed to multiple mechanisms. BCP may stimulate erythropoiesis via hypoxia‐inducible factor pathways (Hajiaqaei et al. 2024; Jacquemin et al. 2024; Shija et al. 2024; Zhang et al. 2024) or may promote nitric oxide production through its arginine composition (Suzuki et al. 2019), improving vascular function and reducing lactic acid accumulation. Additionally, the amino acids and bioactive peptides in BCP may activate antioxidant enzyme systems and reduce oxidative stress, further delaying fatigue onset and enhancing exercise capacity (Chen et al. 2022; Kwon et al. 2021; Ma et al. 2020; Zdzieblik et al. 2015; Zhou et al. 2024). These findings suggest that BCP is a promising, safe, and effective nutritional ingredient that can improve exercise performance in HH environments.

## 5 Conclusions

This study systematically evaluated the amino acid composition, physicochemical properties, and antioxidant activity of BCP, alongside their physiological effects under HH conditions. The animal experiments showed that BCP supplementation significantly enhanced hypoxia tolerance and anti‐fatigue capacity in mice, as evidenced by an 8‐fold increase in rotarod time under HH conditions. Similarly, human trials conducted at a simulated altitude of 3600 m revealed that BCP improved resting oxygen saturation, reduced HR, and significantly enhanced hypoxia tolerance and anti‐fatigue capacity during the submaximal exercise test. These findings highlight BCP's dual potential as an antioxidant and a hypoxia‐tolerance enhancer, making it a valuable functional ingredient for addressing oxidative stress and fatigue in HH environments. BCP holds promise for applications in nutritional products targeting athletes, fitness enthusiasts, and individuals exposed to high‐altitude or hypoxic conditions. Further research on its mechanisms of action and long‐term effects will provide additional insights into its functional potential.

## Author Contributions

**Rui Zhang:** formal analysis (equal), software (equal), validation (equal), visualization (equal), writing – original draft (equal). **Zi‐Xian Fu:** investigation (equal), software (equal), validation (equal), visualization (equal). **Jun‐Bin Xiong:** investigation (supporting), validation (equal). **Wei‐Hong Guo:** investigation (equal), resources (equal). **Wen‐Pu Shi:** resources (equal). **Bin Jia:** project administration (supporting), resources (equal). **Jun‐Ling Shi:** project administration (supporting). **Da‐Chuan Yin:** conceptualization (lead), funding acquisition (lead), project administration (lead), supervision (lead), writing – review and editing (lead).

## Ethics Statement

The experiments conducted in this study are compliant with the existing national legislation. All animal experiments strictly adhered to international ethical guidelines, and human trials adhered to the guidelines outlined in the WMA Declaration of Helsinki and CIOMS International Ethical Guidelines for Human Biomedical Research, and received approval from the Northwestern Polytechnical University Medical and Laboratory Animal Ethics Committee (No. 202202038, May 9th, 2022). Prior to participation, all participants provided informed consent after comprehensively understanding the experimental procedures and protocols.

## Conflicts of Interest

The authors declare no conflicts of interest.

## Supporting information


Appendix S1.

## Data Availability

All relevant data are included within the manuscript. Any inquiries concerning the findings of this study may be directed to the corresponding author upon request.
