# Supplementary material for: Bovine Collagen Peptide Improves Hypoxia Tolerance and Anti‐Fatigue Capacity in Hypobaric Hypoxic Environments: A Combined Animal and Human Study
Source: Food Sci Nutr. 2025 May 15;13(5):e70278. doi: 10.1002/fsn3.70278 (PMC12079017; doi:10.1002/fsn3.70278)
Supplement: Supplementary file 1 — Appendix S1. [file FSN3-13-e70278-s001.doc]

**Supplementary Information**

**Bovine Collagen Peptide Improves Hypoxia Tolerance and Anti-Fatigue Capacity in Hypobaric Hypoxic Environments: A Combined Animal and Human Study**


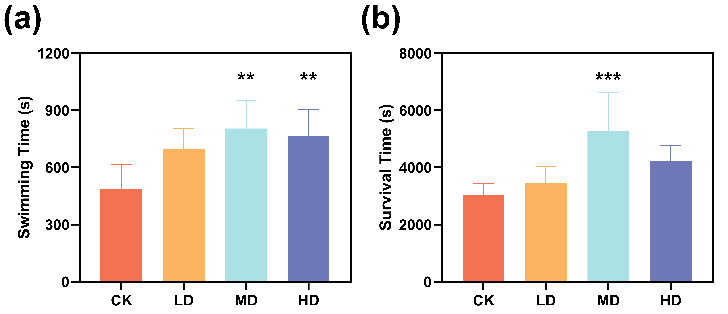


**Fig S1**. Effects of different doses of BCP on anti-fatigue and hypoxic tolerance in mice. (a) Exhaustion time of mice swimming with loaded weights under normal pressure and oxygen conditions; (b) Survival time of mice in normobaric hypoxia test.

**Table S1: Analysis of Amino Acid Composition in BCP**

|  | Retention Time (min) | Peak Area (mV.s) | Concentration (mg/g) | Name | Wavelength  (nm) |
| --- | --- | --- | --- | --- | --- |
| 1 | 8.768 | 2418.433 | 58.255 | Asp | 570 |
| 2 | 10.567 | 951.607 | 20.306 | Thr |
| 3 | 11.505 | 1797.987 | 33.502 | Ser |
| 4 | 13.84 | 4159.177 | 116.948 | Glu |
| 5 | 20.1 | 17930.735 | 207.221 | Gly |
| 6 | 21.113 | 5224.047 | 94.226 | Ala |
| 7 | 22.191 | 37.783 | 1.091 | Cys |
| 8 | 22.843 | 1195.448 | 24.724 | Val |
| 9 | 24.82 | 365.267 | 7.712 | Met |
| 10 | 26.14 | 515.449 | 12.544 | Ile |
| 11 | 27.127 | 1353.186 | 27.996 | Leu |
| 12 | 30.047 | 114.071 | 3.324 | Tyr |
| 13 | 30.928 | 723.523 | 18.75 | Phe |
| 14 | 36.079 | 229.45 | 5.44 | His |
| 15 | 37.209 | 1817.473 | 42.13 | Lys |
| 16 | 45.927 | 2484.381 | 76.83 | Arg |
| 17 | 15.195 | 922.393 | 134.145 | Pro | 440 |

**Table S2**: Effect of BCP on complete blood count in mice

| **Indicators** | **Control** | **BCP** |
| --- | --- | --- |
| WBC (10^9^/L) | 6.96 ± 5.61 | 5.23 ± 1.28 |
| NEUT (%) | 9.86 ± 3.93 | 8.33 ± 3.72 |
| LYMPH (%) | 89.45 ± 52.87 | 76.35 ± 5.85 |
| MONO (%) | 2.62 ± 4.53 | 0.25 ± 0.17 |
| EO (%) | 2.42 ± 4.26 | 0.15 ± 0.17 |
| BASO (%) | 39.66 ± 41.15 | 14.93 ± 3.03 |
| NEUT (10^9^/L) | 4.32 ± 5.42 | 0.43 ± 0.22 |
| LYMPH (10^9^/L) | 2.02 ± 0.61 | 4.00 ± 1.12 |
| MONO (10^9^/L) | 2.02 ± 4.47 | 0.02 ± 0.01 |
| EO (10^9^/L) | 2.01 ± 4.46 | 0.01 ± 0.01 |
| BASO (10^9^/L) | 4.61 ± 5.32 | 0.78 ± 0.22 |
| RBC (10^12^/L) | 9.10 ± 0.97 | 10.33 ± 0.22 |
| HCT (%) | 49.70 ± 4.88 | 53.48 ± 2.22 |
| MCV (fL) | 55.60 ± 2.50 | 52.00 ± 1.58 |
| RDW-SD (fL) | 32.92 ± 5.35 | 28.55 ± 1.20 |
| RDW-CV (%) | 20.40 ± 1.93 | 20.40 ± 0.67 |
| HGB (g/L) | 133.00 ± 17.12 | 146.50 ± 4.20 |
| MCH (pg) | 14.74 ± 0.33 | 14.20 ± 0.32 |
| MCHC (g/L) | 265.60 ± 8.11 | 273.25 ± 5.32 |
| PLT (10^9^/L) | 1239.40 ± 402.02 | 1025.50 ± 364.43 |
| MPV (fL) | 6.70 ± 0.12 | 6.78 ± 0.30 |
| PCT (%) | 0.70 ± 0.14 | 0.70 ± 0.27 |
| PDW (fL) | 6.54 ± 0.11 | 6.75 ± 0.17 |
| P-LCR (%) | 4.42 ± 0.63 | 5.05 ± 2.45 |

WBC: White Blood Cell; NEUT: Neutrophilic Granulocyte; LYMPH: Lymphocyte; MONO: Monocyte; EO: Eosinophilic; BASO: Basophil; RBC: Red Blood Cell; HCT: Hematokrit; MCV: Mean Corpuscular Volume; RDW-SD: Red Blood Cell Distribution Width-Standard Deviation; RDW-CV: Red Cell Distribution Width Coefficient of Variation; HGB: Hemoglobin; MCH: Mean Corpuscular Hemoglobin; MCHC: Mean Corpuscular Hemoglobin Concentration; PLT: Platelet; MPV: Mean Platelet Volume; PCT: Plateletcrit; PDW: Platelet Distribution Width; P-LCR: Platelet-Large Cell Ratio

**Table. S3** Complete blood count of placebo participants before and after ingestion

| **Indicators** | **Placebo-before** | **Placebo-after** |
| --- | --- | --- |
| WBC (10^9^/L) | 5.48 ± 0.81 | 5.48 ± 0.44 |
| NEUT (%) | 50.90 ± 6.35 | 47.42 ± 8.64 |
| LYMPH (%) | 38.43 ± 7.49 | 41.00 ± 977 |
| MONO (%) | 7.40 ± 2.03 | 7.77 ± 1.40 |
| EO (%) | 2.72 ± 1.67 | 3.13 ± 1.51 |
| BASO (%) | 0.55 ± 0.32 | 0.72 ± 0.24 |
| NEUT (10^9^/L) | 2.80 ± 0.58 | 2.58 ± 0.42 |
| LYMPH (10^9^/L) | 2.10 ± 0.50 | 2.26 ± 0.61 |
| MONO (10^9^/L) | 0.40 ± 0.11 | 0.42 ± 0.06 |
| EO (10^9^/L) | 0.16 ± 0.11 | 0.18 ± 0.09 |
| BASO (10^9^/L) | 0.03 ± 0.02 | 0.04 ± 0.01 |
| RBC (10^12^/L) | 4.56 ± 0.41 | 4.55 ± 0.50 |
| HCT (%) | 39.28 ± 3.61 | 39.35 ± 4.06 |
| MCV (fL) | 86.22 ± 4.95 | 86.75 ± 5.22 |
| RDW-SD (fL) | 39.62 ± 1.15 | 39.37 ± 1.07 |
| RDW-CV (%) | 12.42 ± 0.70 | 12.32 ± 0.81 |
| HGB (g/L) | 136.50 ± 13.88 | 132.33 ± 16.01 |
| MCH (pg) | 29.58 ± 2.01 | 29.15 ± 2.03 |
| MCHC (g/L) | 343.00 ± 9.65 | 336.00 ± 8.20 |
| PLT (10^9^/L) | 252.33 ± 40.13 | 243.00 ± 32.56 |
| MPV (fL) | 10.73 ± 0.91 | 10.82 ± 0.90 |
| PCT (%) | 0.27 ± 0.03 | 0.26 ± 0.03 |
| PDW (fL) | 12.83 ± 2.91 | 13.08 ± 2.33 |
| P-LCR (%) | 30.03 ± 7.58 | 30.80 ± 7.13 |

WBC: White Blood Cell; NEUT: Neutrophilic Granulocyte; LYMPH: Lymphocyte; MONO: Monocyte; EO: Eosinophilic; BASO: Basophil; RBC: Red Blood Cell; HCT: Hematokrit; MCV: Mean Corpuscular Volume; RDW-SD: Red Blood Cell Distribution Width-Standard Deviation; RDW-CV: Red Cell Distribution Width Coefficient of Variation; HGB: Hemoglobin; MCH: Mean Corpuscular Hemoglobin; MCHC: Mean Corpuscular Hemoglobin Concentration; PLT: Platelet; MPV: Mean Platelet Volume; PCT: Plateletcrit; PDW: Platelet Distribution Width; P-LCR: Platelet-Large Cell Ratio

Placebo-before: before placebo supplementation

Placebo-after: after placebo supplementation.

Mean ± SD: Mean ± Standard Deviation.

**Table. S4** Blood biochemical indicatorss of placebo participants before and after ingestion

| **Indicators** | **Placebo-before** | **Placebo-after** |
| --- | --- | --- |
| TBIL (μmol/L) | 16.47 ± 6.20 | 15.53 ± 9.21 |
| DBIL (μmol/L) | 3.23 ± 0.56 | 2.97 ± 0.90 |
| IBIL (μmol/L) | 13.23 ± 5.74 | 12.57 ± 8.43 |
| TP (g/L) | 73.40 ± 2.18 | 71.28 ± 1.67 |
| ALB (g/L) | 45.70 ± 2.80 | 44.13 ± 0.93 |
| GLB (g/L) | 27.70 ± 3.66 | 27.15 ± 1.52 |
| A/G | 1.68 ± 0.28 | 1.63 ± 0.10 |
| ALT (U/L) | 10.53 ± 3.81 | 9.70 ± 4.31 |
| AST (U/L) | 15.93 ± 2.27 | 18.72 ± 3.98 |
| AST/ALT | 1.63 ± 0.42 | 2.17 ± 0.70 |
| ALP (U/L) | 45.52 ± 7.67 | 55.80 ± 7.53 |
| GGT (U/L) | 10.18 ± 1.87 | 9.30 ± 1.37 |
| CHE (U/L) | 6553.17 ± 844.05 | 6424.83 ± 1007.17 |
| CHOL (mmol/L) | 3.51 ± 0.46 | 3.40 ± 0.43 |
| TG (mmol/L) | 0.66 ± 0.08 | 0.46 ± 0.15 |
| HDL (mmol/L) | 1.32 ± 0.29 | 1.27 ± 0.28 |
| LDL (mmol/L) | 1.89 ± 0.39 | 1.93± 0.35 |
| UA (μmol/L) | 345.75 ± 85.77 | 340.93 ± 77.81 |
| BUN (mmol/L) | 3.95 ± 0.99 | 4.05 ± 0.93 |
| SCR (μmol/L) | 59.43 ± 9.72 | 65.43 ± 8.94 |
| GLU (mmol/L) | 4.67 ± 0.33 | 4.34 ± 0.37 |

TBIL: Total Bilirubin; DBIL: Direct Bilirubin; IBIL: Indirect Bilirubin; TP: Total Protein; ALB: Albumin; GLB: Globulin; ALT: Alanine Aminotransferase; AST: Aspartate Transaminase; ALP: Alkaline Phosphatase; GGT: Gamma-glutamyltransferase; CHE: Cholinesterase; CHOL: Total Cholesterol; TG: Triglyceride; HDL: High Density Lipoprotein; LDL: Low Density Lipoprotein; UA: Uric Acid; BUN: Blood Urea Nitrogen; SCR: Serum Creatinine; GLU: Glucose

Placebo-before: before placebo supplementation

Placebo-after: after placebo supplementation.

Mean ± SD: Mean ± Standard Deviation.

**Table. S5** Complete blood count of BCP participants before and after ingestion

| **Indicators** | **BCP-before** | **BCP-after** |
| --- | --- | --- |
| WBC (10^9^/L) | 5.63 ± 0.83 | 5.80 ± 0.71 |
| NEUT (%) | 50.97 ± 4.04 | 50.07 ± 5.48 |
| LYMPH (%) | 39.95 ± 3.26 | 40.85 ± 5.63 |
| MONO (%) | 7.13 ± 0.88 | 7.05 ± 1.45 |
| EO (%) | 1.43 ± 0.88 | 1.58 ± 0.79 |
| BASO (%) | 0.52 ± 0.21 | 0.45 ± 0.19 |
| NEUT (10^9^/L) | 2.89 ± 0.58 | 2.91 ± 0.53 |
| LYMPH (10^9^/L) | 2.24 ± 0.25 | 2.36 ± 0.37 |
| MONO (10^9^/L) | 0.40 ± 0.08 | 0.41 ± 0.10 |
| EO (10^9^/L) | 0.08 ± 0.04 | 0.09 ± 0.04 |
| BASO (10^9^/L) | 0.03 ± 0.02 | 0.03 ± 0.01 |
| RBC (10^12^/L) | 4.89 ± 0.50 | 5.04 ± 0.51 |
| HCT (%) | 42.68 ± 4.49 | 44.10 ± 4.63 |
| MCV (fL) | 87.25 ± 2.62 | 87.53 ± 2.17 |
| RDW-SD (fL) | 38.25 ± 1.32 | 38.90 ± 1.07 |
| RDW-CV (%) | 12.18 ± 0.47 | 11.88 ± 0.40 |
| HGB (g/L) | 146.00 ± 14.55 | 150.17 ± 13.59 |
| MCH (pg) | 29.87 ± 0.87 | 29.85 ± 1.07 |
| MCHC (g/L) | 342.33 ± 5.47 | 341.17 ± 8.33 |
| PLT (10^9^/L) | 217.33 ± 12.32 | 221.83 ± 27.91 |
| MPV (fL) | 10.17 ± 0.45 | 10.47 ± 0.54 |
| PCT (%) | 0.22 ± 0.01 | 0.23 ± 0.03 |
| PDW (fL) | 11.75 ± 1.01 | 12.40 ± 1.74 |
| P-LCR (%) | 26.20 ± 3.28 | 28.73 ± 4.71 |

WBC: White Blood Cell; NEUT: Neutrophilic Granulocyte; LYMPH: Lymphocyte; MONO: Monocyte; EO: Eosinophilic; BASO: Basophil; RBC: Red Blood Cell; HCT: Hematokrit; MCV: Mean Corpuscular Volume; RDW-SD: Red Blood Cell Distribution Width-Standard Deviation; RDW-CV: Red Cell Distribution Width Coefficient of Variation; HGB: Hemoglobin; MCH: Mean Corpuscular Hemoglobin; MCHC: Mean Corpuscular Hemoglobin Concentration; PLT: Platelet; MPV: Mean Platelet Volume; PCT: Plateletcrit; PDW: Platelet Distribution Width; P-LCR: Platelet-Large Cell Ratio

BCP-before: before BCP supplementation

BCP-after: after BCP supplementation.

Mean ± SD: Mean ± Standard Deviation.

**Table. S6** Blood biochemical indicatorss of BCP participants before and after ingestion

| **Indicators** | **BCP-before** | **BCP-after** |
| --- | --- | --- |
| TBIL (μmol/L) | 14.92 ±4.95 | 14.42 ± 5.91 |
| DBIL (μmol/L) | 3.32 ±0.56 | 3.77 ± 1.03 |
| IBIL (μmol/L) | 11.60 ± 4.63 | 10.65 ± 5.51 |
| TP (g/L) | 72.2 ± 3.09 | 73.05 ± 2.36 |
| ALB (g/L) | 45.17 ± 1.99 | 45.95 ± 1.31 |
| GLB (g/L) | 27.03 ±2.20 | 27.10 ± 2.65 |
| A/G | 1.68 ±0.16 | 1.71 ± 0.20 |
| ALT (U/L) | 20.25 ± 16.09 | 15.10 ± 12.27 |
| AST (U/L) | 16.60 ± 4.64 | 16.18 ± 2.69 |
| AST/ALT | 1.05 ± 0.41 | 1.60 ± 0.90 |
| ALP (U/L) | 59.93 ± 12.42 | 55.17 ± 9.14 |
| GGT (U/L) | 23.55 ± 14.30 | 20.82 ± 11.65 |
| CHE (U/L) | 8579.83 ± 2956.74 | 8561.33 ± 2791.83 |
| CHOL (mmol/L) | 4.67 ± 1.08 | 4.43 ± 0.83 |
| TG (mmol/L) | 0.90 ± 0.60 | 0.69 ± 0.39 |
| HDL (mmol/L) | 1.27 ± 0.15 | 1.26±0.22 |
| LDL (mmol/L) | 2.99 ± 0.98 | 2.85 ± 0.85 |
| UA (μmol/L) | 406.40 ± 86.60 | 408.08 ± 76.51 |
| BUN (mmol/L) | 3.85 ± 0.29 | 3.62 ± 0.53 |
| SCR (μmol/L) | 71.45 ± 10.00 | 71.05± 7.00 |
| GLU (mmol/L) | 4.97 ± 0.34 | 4.58 ± 0.25 |

TBIL: Total Bilirubin; DBIL: Direct Bilirubin; IBIL: Indirect Bilirubin; TP: Total Protein; ALB: Albumin; GLB: Globulin; ALT: Alanine Aminotransferase; AST: Aspartate Transaminase; ALP: Alkaline Phosphatase; GGT: Gamma-glutamyltransferase; CHE: Cholinesterase; CHOL: Total Cholesterol; TG: Triglyceride; HDL: High Density Lipoprotein; LDL: Low Density Lipoprotein; UA: Uric Acid; BUN: Blood Urea Nitrogen; SCR: Serum Creatinine; GLU: Glucose

Placebo: Placebo group, BCP: Bovine Collagen Peptide group

BCP-before: before BCP supplementation

BCP-after: after BCP supplementation.

Mean ± SD: Mean ± Standard Deviation.
